# Disappearance and appearance of an indigestible marker in feces from growing pigs as affected by previous- and current-diet composition

**DOI:** 10.1186/s40104-017-0161-9

**Published:** 2017-04-01

**Authors:** Brandy M. Jacobs, John F. Patience, Merlin D. Lindemann, Kenneth J. Stalder, Brian J. Kerr

**Affiliations:** 1 Department of Animal Science, Iowa State University, Ames, 50010 USA; 2 Department of Animal and Food Sciences, University of Kentucky, Lexington, 40546 KY USA; 3 USDA-ARS-National Laboratory for Agricultural and the Environment, Ames, 50010 IA USA

**Keywords:** Adaptation, Digestibility, Fiber, Indigestible marker, Pig

## Abstract

**Background:**

Indigestible markers are commonly utilized in digestion studies, but the complete disappearance or maximum appearance of a marker in feces can be affected by diet composition, feed intake, or an animal’s BW. The objectives of this study were to determine the impact of previous (Phase 1, P1) and current- (Phase 2, P2) diet composition on marker disappearance (Cr) and appearance (Ti) in pigs fed 3 diets differing in NDF content.

**Results:**

When pigs were maintained on the 25.1, 72.5, and 125.0 g/kg NDF diets, it took 5.1, 4.1, and 2.5 d, respectively, for Cr levels to decrease below the limit of quantitation; or 4.6, 3.7, or 2.8 d, respectively, for Ti to be maximized. These effects were not, however, independent of the previous diet as indicated by the interaction between P1 and P2 diets on fecal marker concentrations (*P* < 0.01). When dietary NDF increased from P1 to P2, it took less time for fecal Cr to decrease or fecal Ti to be maximized (an average of 2.5 d), than if NDF decreased from P1 to P2 where it took longer for fecal Cr to decrease or fecal Ti to be maximized (an average of 3.4 d).

**Conclusions:**

Because of the wide range in excretion times reported in the literature and improved laboratory methods for elemental detection, the data suggests that caution must be taken in considering dietary fiber concentrations of the past and currently fed diets so that no previous dietary marker addition remains in the digestive tract or feces such that a small amount of maker is present to confound subsequent experimental results, and that marker concentration have stabilized when these samples are collected.

## Background

Indigestible markers are commonly used in animal nutrition studies to calculate digestibility coefficients, with chromic oxide, titanium dioxide, and acid insoluble ash being the most common in swine research [1]. Physiological aspects associated with gastric emptying or rate of passage are complex and affected by a variety of factors [2, 3]. Rate of passage can be affected by BW [4], feed intake level [5], dietary fiber type and level [6–8], particle size [9], and genetics [10]. In addition, rates of passage in the gastrointestinal tract are not consistent, being pulsatile over time [11, 12]. The appearance of the first marker peak is relatively consistent at the terminal ileum of pigs, occurring approximately 6 h following a meal, dropping to minimum levels 24 h post-meal [13]. In contrast, digesta flow through the hind gut is longer and more variable, where mean transit times through the entire digestive tract have been reported to be less than 50 h [7] to over 100 h [6]. Imbeath et al. [13] reported that 4 d was needed before marker concentrations were near zero after marker withdrawal, while others [14, 15] have reported that their appearance in the feces is stabilized 4 to 5 d after feeding.

Currently, there is no standard time for pigs to be adapted to a diet, a specific number of days an animal should be sampled, or the number of days between collection periods in swine research utilizing inert markers. As a consequence, the objectives of this study were to: 1) determine the impact of previous (P1) and currently-fed (P2) diet composition on the complete disappearance P2 marker (Cr) and 2) determine the impact of previous and currently-fed diet composition on the complete appearance of P2 marker (Ti) in growing pigs fed diets differing in fiber content.

## Methods

The experiment was conducted under protocols approved by the University of Kentucky Institutional Animal Care and Use Committee.

### Feeding management

Diets (Table 1) were formulated to contain varying levels of NDF through the utilization of dehulled, degermed corn (DDC), corn (C), soybean meal (S), and distillers dried grains with solubles (DDGS). Diets were formulated to meet requirements relative to NRC (1998) recommendations. The same diet composition was used in each of 2 phases, with Phase-1 (P1) diets utilizing chromic oxide and Phase-2 diets (P2) utilizing titanium dioxide, each added at 5.0 g/kg to the complete diet at the time of mixing to determine fecal marker concentrations. Two different inert markers were utilized to distinguish the feces originating from the diets consumed during P1 to the feces originating from the diet consumed in P2. This allows for the comparing the disappearance of the marker used in P1 and the appearance of the marker used in P2. This also prevents any potential contamination of the marker in the digestive tract in P1 with that of P2, which would have prevented the pre-planned comparisons of marker disappearance and appearance during the P2 period relative to a diet change. Pigs were provided ad libitum access to feed and water throughout the experiment.

**Table 1:** Composition of Phase-1 and Phase-2 diets, as-fed basis^a^

	DDC	CS	DDGS
Ingredient, g/kg			
Corn	–	784.0	567.0
Soybean meal	180.0	180.0	150.0
Dehulled, degermed corn	781.9	–	–
Dried distillers grains with solubles	–	–	250.0
Soybean oil	5.0	5.0	5.0
L-Lysine · HCl	1.1	–	–
Dicalcium phosphate	8.5	7.0	1.5
Limestone	7.0	7.5	10.0
Sodium chloride	5.0	5.0	5.0
Vitamin premix^b^	0.5	0.5	0.5
Trace mineral premix^c^	0.5	0.5	0.5
Marker^d^	5.0	5.0	5.0
Clay^e^	5.0	5.0	5.0
Antibiotic^f^	0.5	0.5	0.5
Calculated composition, g/kg unless otherwise noted			
Calcium	5.0	5.0	5.0
Crude fat,	12.0	41.0	53.0
Crude protein	142.7	150.6	187.6
Lysine	7.5	7.5	7.5
Metabolizable energy, kcal/kg	3,293	3,332	3,193
NDF	45.0	91.0	154.0
Phosphorus	3.4	4.7	4.8
Sulfur	1.0	1.8	2.1
Analyzed composition, g/kg unless otherwise noted^g^			
Crude fat	12.8	37.2	47.7
Crude protein	132.5	160.0	197.5
Gross energy, kcal/kg	3,770	3,973	4,131
NDF	25.1	72.5	125.0
Phosphorus	2.8	4.6	4.9
Sulfur	1.7	2.0	3.2

^a^ *Abbreviations*: *DDC* dehulled, degermed corn, *CS* corn, soybean meal, *DDGS* distillers dried grains with solubles

^b^Supplied per kilogram of diet: vitamin A, 6,600 IU; vitamin D_3_, 880 IU; vitamin E, 44 IU; vitamin K (menadione sodium bisulfate complex), 6.4 mg; thiamin, 4.0 mg; riboflavin, 8.8 mg; pyridoxine, 4.4 mg; vitamin B_12_, 33 μg; folic acid, 1.3 mg; niacin, 44 mg; pantothenic acid, 22 mg; and D-biotin, 0.22 mg

^c^Supplied per kilogram of diet: Zn, 131 mg as ZnO; Fe, 131 mg as FeSO_4_ · H_2_O; Mn 45 mg, as MnO; Cu, 13 mg as CuSO_4_ · 5H_2_O; I, 1.5 mg as CaI_2_O_6_; Co, 0.23 mg as CoCO_3_; and Se, 0.28 mg as Na_2_O_3_Se

^d^The addition of 0.5%, Cr_2_O_3_ (≥98% purity; Elementis Chromium LP, Corpus Christi, TX) represents an addition of 3.35 mg Cr/g diet; averaged across diets, the analyzed content equaled 2.76 mg Cr/kg diet (Phase-1). The addition of 0.5% TiO2 (99% purity, Tronox Pigments GmBH, Krefield, Germany) represents an addition of 2.97 mg titanium/g diet; averaged across diets, the analyzed content equaled 2.89 mg titanium/kg diet (Phase-2)

^e^AB-20 (Prince Agriproducts, Quincy, IL)

^f^Tylan-40 supplied 44 mg/kg of diet (Elanco, Greenfield, IN)

^g^Diets were analyzed at the USDA-ARS (Ames, IA), except for phosphorus which was analyzed by SDK Labs (Hutchison, KS)

### Pig management and collections

**Fig. 1:**
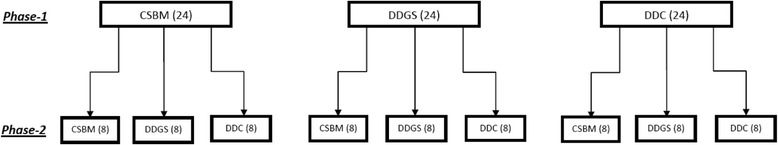
Allotment of 72 crossbred barrows into Phase-1 (d 0 to 14) and Phase-2 (d 14 to 28) diets. DDC = dehulled, degermed corn; CSBM = corn, soybean meal; and DDGS = distillers dried grains with solubles. Numbers in parentheses represent the initial number of pigs per treatment

Seventy two crossbred barrows [(Yorkshire × Landrace × Duroc) × Chester White] were individually penned and randomly assigned to 1 of 3 dietary treatments. Pigs were initially separated into 3 treatment groupings of 24 pigs (d-0; 59.2 kg BW, 4.81 kg SD) and fed ad libitum P1 diets for 14 d (d-14; 75.4 kg BW, 5.71 kg SD) and then randomly reassigned within P1 dietary treatment into 1 of 3 P2 dietary treatments, and fed ad libitum an additional 14 days (d-28; 88.6 kg BW, 5.46 kg SD), resulting in 9 treatment groups of 8 pigs each (Fig. 1). For each pig and each day during P2 (d-14 through d-28), freshly excreted fecal samples (samples either from the anus or after just dropping on the floor—but not contaminated with feed or existing feces) were collected into plastic containers and placed into a −20 °C freezer until analyzed. Samples were collected from 0700 to 1200 h on each collection day to be consistent in sample collection during the 14 d and to ensure an adequate sample size for subsequent analysis.

### Chemical analysis

Prior to analysis, fecal samples were dried in a forced-air oven at 70 °C for 48 h prior to grinding. Feed and fecal samples were ground through a 1-mm screen before composition was determined. Chromic oxide in feces was analyzed for Cr at a commercial laboratory (SDK Labs, Hutchinson, KS) by inductively coupled plasma spectroscopy (Ultima 2; Horiba Jobin-Yvon Inc., Edison, NJ) according to standard method (3120B; American Public Health Association, 1992) with a limit of quantitation (LOQ) of 0.3 mg Cr/kg sample. Titanium dioxide in feces was analyzed for Ti by digesting the samples in sulfuric acid and hydrogen peroxide and subsequent absorbance was measured using a UV spectrophotometer (Method 988.05; [16]), with a LOQ of 6 mg Ti/kg sample (USDA-ARS, Ames, IA). Because reporting a zero (0) for data below the LOQ artificially skews analytical values to 0, any value analyzed below the LOQ but above the limit of detection (values above the blank value used in standard curve assays), was assumed to be 50% of the LOQ, which is common in the chemical analysis industry.

### Calculations and statistical analysis

All data were analyzed using mixed model methods using PROC MIXED (SAS Inst., Cary, NC). The model included P1 dietary treatment, P2 dietary treatment, and P1 × P2 dietary interaction as fixed effects. For fecal Cr disappearance or Ti appearance during P2 as affected by the P1 diet, both d-14 BW and d-8 to 14 ADFI were used as model linear covariates [17]. However, for fecal Cr disappearance or Ti appearance during P2 as affected by the P2 diet, both d-14 BW and d-15 to 21 ADFI were used as model linear covariates. Only BW was utilized as a model linear covariate for the interaction between P1 and P2 diet. Regardless of significance, BW (which was often significant) and ADFI (which was often not significant) were retained in the model. Pig within treatment was included as a random effect in all models. Means are reported as least square means with fecal Cr or Ti concentrations plotted over time to show the disappearance of Cr and appearance of Ti during P2, relative to P1 or P2 diet composition. Fecal Cr or Ti concentrations for the final 7 d in P2, are not shown because there were no changes in fecal Cr or Ti during that time period or the levels were below LOQ. Estimates of the number of days for fecal Cr to decrease to the LOQ or for fecal Ti to reach 95% of its maximum value for each P1 × P2 combination was determined fitting a 4 parameter sigmoidal logistic function [$\left( y= D+\frac{\left( A - D\right)}{1 + {\left(\frac{x}{C}\right)}^B}\right)$; where *x* = collection time, *y* = the response value (Cr or Ti concentration), A = minimum point in the line B = slope in the middle of the curve, C = point of inflection, D = maximum of the line; Microsoft Excel 2010] to the overall treatment means.

## Results

### Dual marker recovery

A critical factor for the present study was that analysis of Cr and Ti in the same diet would not interfere with the analysis of either element. To evaluate this, 3 separate corn-soybean meal diets were mixed which contained either 5 g chromic oxide/kg diet (Diet 1), 5 g titanium dioxide/kg diet (Diet 2), or both 5 g chromic oxide and 5 g titanium dioxide/kg diet (Diet 3). Although Cr analysis was lower than expected averaging 2,600 mg Cr/kg diet versus an expected level of 3,395 mg Cr/kg diet (5,000 mg Cr_2_O_3_ added × 99.3% purity × 684 g/kg Cr), it did not differ whether added either alone (Diet 1) or with titanium dioxide (Diet 3), Table 2. Titanium in Diets 2 and 3 averaged 2,502 mg/kg diet after subtracting out the apparent background Ti level noted in Diet 1. This too was lower than the expected value of 2,970 mg Ti/kg diet (5,000 mg TiO_2_ added × 99.0% purity × 600 g/kg Ti). There were slight differences in Ti levels between Diet 2 (2,530 mg Ti/kg diet) with only TiO_2_ added, and Diet 3 (2,884 mg Ti/kg diet) when both and Cr_2_O_3_ and TiO_2_.

**Table 2:** Marker concentrations in corn-soybean meal diet containing either titanium dioxide, chromic oxide, or both titanium dioxide and chromic oxide^a^

	Titanium, mg/kg diet	Chromium, mg/kg diet
*Diet 1-Cr*_*2*_ *O*_*3*_		
Sample 1	240	2,700
Sample 2	173	2,400
Sample 3	246	2,900
Sample 4	161	2,400
Mean	205	2,600
SD	44	245
CV	21.6	9.4
*Diet 2-TiO*_*2*_		
Sample 1	2,491	<0.01
Sample 2	2,529	<0.01
Sample 3	2,635	<0.01
Sample 4	2,465	<0.01
Mean	2,530	–
SD	75	–
CV	3.0	–
*Diet 3-Cr*_*2*_ *O*_*3*_ *and TiO*_*2*_		
Sample 1	2,942	2,400
Sample 2	2,831	2,500
Sample 3	2,995	2,800
Sample 4	2,768	2,700
Mean	2,884	2,600
SD	103	183
CV	3.6	7.0

^a^The addition of 5,000 mg Cr_2_O_3_ added × 99.3% purity × 684 g/kg Cr would result in an expected level of 3,395 mg Cr/kg diet. The addition of 5,000 mg TiO_2_ added × 99.0% purity × 600 g/kg Cr would result in an expected level of 2,970 mg Ti/kg diet

### Fecal Cr disappearance

**Fig. 2:**
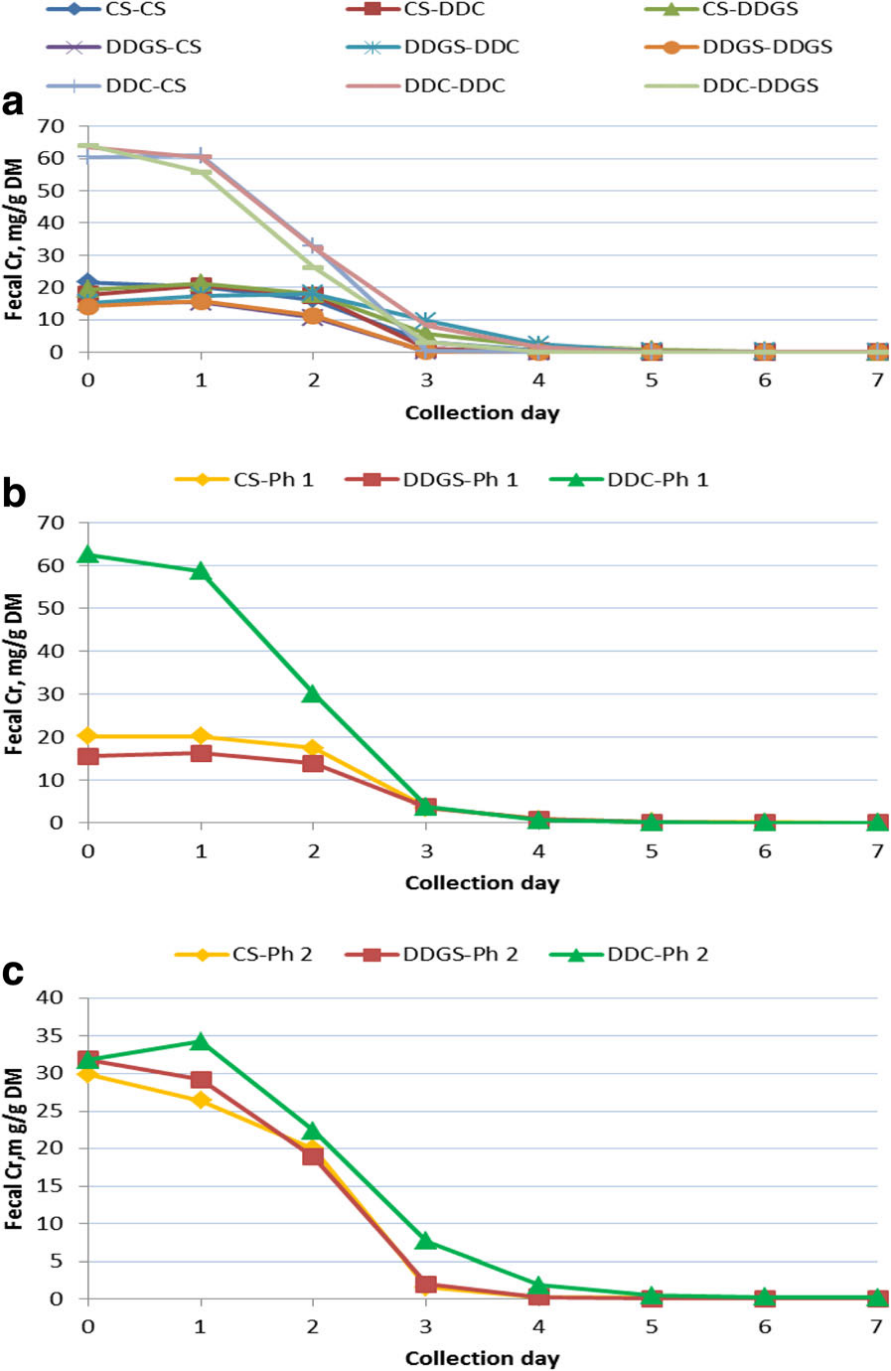
**a** Fecal Cr concentration of growing pigs during Phase 2 as affected by the combination of Phase 1 and Phase 2 diets. Abbreviations: CS, corn-soybean meal based diet; DDGS, corn-soybean meal-distillers dried grains based diet; DDC, dehulled, degermed corn-soybean meal based diet. First abbreviation in legend represents the Phase-1 diet and the second abbreviation in the legend represents the Phase-2 diet. Collection day following change from Phase-1 to Phase-2 diet with d-0 being the day of diet change. **b** Fecal Cr concentration of growing pigs during Phase 2 as affected by Phase 1 diet. Legend abbreviations: CS, corn-soybean meal based diet; DDGS, corn-soybean meal-distillers dried grains based diet; DDC, dehulled, degermed corn-soybean meal based diet. Collection day following change from Phase-1 to Phase-2 diet with d-0 being the day of diet change. **c** Fecal Cr concentration of growing pigs during Phase 2 as affected by Phase 2 diet. Legend abbreviations: CS, corn-soybean meal based diet; DDGS, corn-soybean meal-distillers dried grains based diet; DDC, dehulled, degermed corn-soybean meal based diet. Collection day following change from Phase-1 to Phase-2 diet with d-0 being the day of diet change

Interactions occurred between P1 and P2 diets on fecal Cr disappearance (*P* < 0.01) during P2, with specific values and significance levels listed in Table 3 and graphically depicted in Fig. 2a. Averaged across diet changes, when dietary NDF was increased in the diets fed to pigs from P1 to P2 (i.e., pigs fed the CS diet switched to the DDGS diet and pigs fed the DDC diet switched to either the CS or DDGS diet), it took 2.6 d for each 5 percentage unit increase in NDF for P2 fecal Cr to decrease below the LOQ of 0.3 mg/kg fecal DM. In contrast, when dietary NDF was decreased in the diets fed to pigs from P1 to P2 (i.e., pigs fed the CS diet switched to the DDC diet and pigs fed the DDGS diet switched to either the CS or DDC diet), it took 3.5 d for each 5 percentage unit decrease in NDF for P2 fecal Cr to decrease below the LOQ (Tables 3 and 5). When pigs remained on the same diets from P1 to P2, pigs continually fed the DDC diet containing 25.1 g/kg NDF took 5.1 d for P2 fecal Cr to decrease below the LOQ, while pigs fed the CS diet containing 72.5 g/kg NDF and the DDGS diet containing 125.0 g/kg NDF took 4.1 d and 2.5 d, respectively, for P2 fecal Cr to decrease below the LOQ (Tables 3 and 5).

**Table 3:** Fecal chromium (mg/g fecal DM) of growing pigs during Phase-2 when fed different diets during Phase-1 and Phase-2

Phase × diet combinations		Collection day^b^							
Phase-1	Phase-2	14	15	16	17	18	19	20	21
CS^a^	CS	21.6	20.2	16.2	3.2	0.6	LOQ^c^	LOQ	LOQ
CS	DDGS	17.8	20.6	17.7	1.1	0.4	LOQ	LOQ	LOQ
CS	DDC	19.5	21.4	18.0	5.7	1.8	0.8	0.3	LOQ
DDGS	CS	15.3	15.4	10.7	0.4	LOQ	LOQ	LOQ	LOQ
DDGS	DDGS	14.2	15.8	11.3	0.3	LOQ	LOQ	LOQ	LOQ
DDGS	DDC	15.3	17.5	18.2	9.7	2.4	0.4	0.3	LOQ
DDC	CS	60.4	60.8	32.8	0.6	LOQ	LOQ	LOQ	LOQ
DDC	DDGS	64.0	55.8	26.3	3.0	LOQ	LOQ	LOQ	LOQ
DDC	DDC	63.5	60.4	32.3	8.3	1.5	0.4	LOQ	LOQ
SE		2.47	2.85	4.92	2.21	0.35	0.11	0.04	0.02
Interaction *P* value		0.01	0.01	0.01	0.02	0.01	0.01	0.01	0.01
Main effect of Phase-1 diet									
*Phase-1*	*Phase-2*								
CS	CS/DDGS/DDC	20.1	20.1	17.4	3.5	0.9	0.3	LOQ	LOQ
DDGS	CS/DDGS/DDC	15.5	16.2	13.8	3.6	0.9	LOQ	LOQ	LOQ
DDC	CS/DDGS/DDC	62.5	58.6	30.1	3.8	0.6	LOQ	LOQ	LOQ
SE		1.00	1.45	2.82	1.37	0.36	0.10	0.04	0.03
*P* value		0.01	0.01	0.01	0.98	0.76	0.46	0.28	0.60
Main effect of Phase-2 diet									
*Phase-1*	*Phase-2*								
CS/DDGS/DDC	CS	29.9	26.4	20.0	1.6	0.3	LOQ	LOQ	LOQ
CS/DDGS/DDC	DDGS	31.9	29.2	18.9	2.0	0.3	LOQ	LOQ	LOQ
CS/DDGS/DDC	DDC	31.8	34.3	22.4	7.7	1.9	0.5	0.3	LOQ
SE		5.75	6.08	3.85	1.06	0.21	0.01	0.02	0.01
*P* value		0.95	0.57	0.81	0.01	0.01	0.01	0.01	0.01

^a^ *Abbreviations*: *CS* corn-soybean meal based diet, *DDGS* corn-soybean meal-distillers dried grains based diet, *DDC* dehulled, degermed corn-soybean meal based diet. For Phase-1, ADFI from d 1 to 14 was 2.94, 2.79, and 2.66 kg (SE = 0.07 kg) for pigs fed the DDC, CS, and DDGS diets, respectively. For Phase-2, ADFI from d 14 to 28 was 2.91, 2.84, and 2.63 kg (SE = 0.21 kg) for pigs fed the DDC, CS, and DDGS diets, respectively

^b^Collection day following change from Phase-1 to Phase-2 diet, with d 14 representing the last day of the diet containing the Cr marker was fed

^c^LOQ = limit of quantitation; 0.3 mg Cr/kg; with ½ LOQ used for statistical analysis

The main effect of the P1 diet on P2 fecal Cr concentration is reported in Table 3 and graphically depicted in Fig. 2b. For d-14 and the following 2 d, pigs fed the DDC diet in P1 had a greater P2 fecal Cr concentration of than for pigs fed either the CS or DDGS diet, with pigs fed the CS diet having a higher P2 fecal Cr than pigs fed the DDGS diet for d-14 and d-15, but equal on d-16. No dietary differences were noted thereafter. The main effect of P2 diet on P2 fecal Cr concentration is additionally reported in Table 3 and graphically depicted in Fig. 2c. Phase 2 diets had no impact on P2 fecal Cr concentration among pigs fed the diets for d-14 through d-16, with pigs fed the DDC diet having a higher P2 fecal Cr concentration than pigs fed either the CS or DDGS diets on d-17 and d-18, with no differences in P2 fecal Cr concentration between pigs fed the CS or DDGS diets. Subsequent to d-18, P2 fecal Cr fell below the LOQ for pigs fed the CS or the DDGS diet, but did not decrease below the LOQ in pigs fed the DDC diet until d-21.

### Fecal Ti appearance

**Fig. 3:**
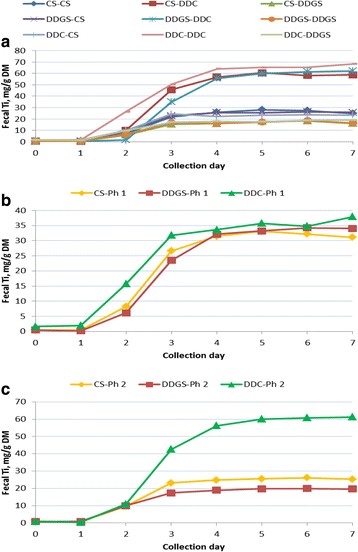
**a** Fecal Ti concentration during Phase 2 as affected by the combination of Phase 1 and Phase 2 diets. Abbreviations: CS, corn-soybean meal based diet; DDGS, corn-soybean meal-distillers dried grains based diet; DDC, dehulled, degermed corn-soybean meal based diet. First abbreviation in legend represents the Phase-1 diet and the second abbreviation in the legend represents the Phase-2 diet. Collection day following change from Phase-1 to Phase-2 diet with d-0 being the day of diet change. **b** Fecal Ti concentration during Phase 2 as affected by Phase 1 diet. Legend abbreviations: CS, corn-soybean meal based diet; DDGS, corn-soybean meal-distillers dried grains based diet; DDC, dehulled, degermed corn-soybean meal based diet. Collection day following change from Phase-1 to Phase-2 diet with d-0 being the day of diet change. **c** Fecal Ti concentration during Phase 2 as affected by Phase 2 diet. Legend abbreviations: CS, corn-soybean meal based diet; DDGS, corn-soybean meal-distillers dried grains based diet; DDC, dehulled, degermed corn-soybean meal based diet. Collection day following change from Phase-1 to Phase-2 diet with d-0 being the day of diet change

Similar to that observed for fecal Cr disappearance, interactions were noted between P1 and P2 diets on fecal Ti appearance during P2, with specific values and significance levels listed in Table 4, and graphically depicted in Fig. 3a. Averaged across diet changes, when dietary NDF was increased in the diets fed to pigs from P1 to P2 (i.e., pigs fed the CS diet switched to the DDGS diet and pigs fed the DDC diet switched to either the CS or DDGS diet), it took 2.4 d for each 5 percentage units increase in NDF for P2 fecal Ti to approach its maximum level. In contrast, when dietary NDF was decreased in the diets fed to pigs from P1 to P2 (i.e., pigs fed the CS diet switched to the DDC diet and pigs fed the DDGS diet switched to either the CS or DDC diet), it took 3.2 d for each 5 percentage units decrease in NDF for P2 fecal Ti to approach its maximum level (Tables 4 and 5). When pigs remained on the same diets from P1 to P2, pigs continually fed the DDC diet containing 25.1 g/kg NDF took 4.6 d for P2 fecal Ti to reach 95% of the maximum level, while pigs fed the CS diet containing 72.5 g/kg NDF and the DDGS diet containing 125.0 g/kg NDF took 3.7 d and 2.8 d, respectively, for P2 fecal Ti to reach 95% of its maximum level (Tables 4 and 5).

**Table 4:** Fecal titanium (mg/g fecal DM) of growing pigs during Phase-2 when fed different diets during Phase-1 and Phase-2

Phase × diet combinations		Collection day^b^							
Phase-1	Phase-2	14	15	16	17	18	19	20	21
CS^a^	CS	LOQ^c^	LOQ	7.3	22.0	25.9	28.1	27.4	24.8
CS	DDGS	LOQ	LOQ	LOQ	15.4	15.9	17.9	18.3	16.1
CS	DDC	LOQ	LOQ	10.1	45.6	56.7	60.6	58.1	58.8
DDGS	CS	LOQ	LOQ	9.2	22.9	25.6	25.5	26.2	25.7
DDGS	DDGS	LOQ	LOQ	6.5	16.8	16.1	17.2	18.7	16.4
DDGS	DDC	LOQ	LOQ	LOQ	34.9	55.6	59.8	61.4	62.1
DDC	CS	LOQ	LOQ	10.0	24.2	22.2	23.2	23.8	23.4
DDC	DDGS	LOQ	LOQ	9.7	17.3	17.9	17.6	18.9	19.1
DDC	DDC	LOQ	LOQ	26.5	50.4	64.0	65.3	65.2	68.3
SE		0.11	0.58	2.82	1.87	2.06	2.16	1.81	2.06
Interaction *P* value		0.01	0.83	0.01	0.01	0.01	0.01	0.01	0.01
Main effect of Phase-1 diet									
*Phase-1*	*Phase-2*								
CS	CS/DDGS/DDC	LOQ	LOQ	8.2	26.6	31.4	33.1	32.2	31.1
DDGS	CS/DDGS/DDC	LOQ	LOQ	6.1	23.5	32.1	33.2	34.2	34.0
DDC	CS/DDGS/DDC	LOQ	LOQ	15.7	31.8	33.6	35.7	34.8	37.9
SE		0.06	0.20	2.62	3.67	4.74	3.99	5.08	5.31
*P* value		0.01	0.01	0.03	0.22	0.93	0.88	0.91	0.61
*Main effect of Phase-2 diet*									
*Phase-1*	*Phase-2*								
CS/DDGS/DDC	CS	LOQ	LOQ	9.9	23.1	24.8	25.5	26.1	25.3
CS/DDGS/DDC	DDGS	LOQ	LOQ	10.0	17.4	18.9	19.8	19.9	19.7
CS/DDGS/DDC	DDC	LOQ	LOQ	10.9	42.6	56.3	60.1	60.8	61.3
SE		0.17	0.36	2.86	1.52	0.70	0.81	0.78	0.76
*P* value		0.79	0.48	0.96	0.01	0.01	0.01	0.01	0.01

^a^ *Abbreviations*: *CS* corn-soybean meal based diet, *DDGS* corn-soybean meal-distillers dried grains based diet, *DDC* dehulled, degermed corn-soybean meal based diet. For Phase-1, ADFI from d 1 to 14 was 2.94, 2.79, and 2.66 kg (SE = 0.07 kg) for pigs fed the DDC, CS, and DDGS diets, respectively. For Phase-2, ADFI from d 14 to 28 was 2.91, 2.84, and 2.63 kg (SE = 0.21 kg) for pigs fed the DDC, CS, and DDGS diets, respectively

^b^Collection day following change from Phase-1 to Phase-2 diet, with d 14 representing the first day of the diet containing the Ti marker was fed

^c^LOQ = limit of quantitation; 6 mg Ti/kg. with ½ LOQ used for statistical analysis

**Table 5:** Sigmoidal response parameters for P2 fecal Cr disappearance and P2 fecal Ti appearance for growing pigs fed different diets during Phase-1 and Phase-2

Phase × diet combinations		NDF ∆	Fecal Cr disappearance,	Fecal Ti appearance,
Phase-1	Phase-2	% Units^b^	d reach limit of quantitation^c^	d to reach 95% maximum^d^
CS^a^	CS	0	4.1	3.7
CS	DDGS	+5.25	3.3	3.5
CS	DDC	−4.74	4.9	3.9
DDGS	CS	−5.25	3.1	3.5
DDGS	DDGS	0	2.5	2.8
DDGS	DDC	−9.99	4.8	4.2
DDC	CS	+4.74	2.6	2.2
DDC	DDGS	+9.99	3.8	3.2
DDC	DDC	0	5.1	4.6

^a^ *Abbreviations*: *CS* corn-soybean meal based diet, *DDGS* corn-soybean meal-distillers dried grains based diet, *DDC* dehulled, degermed corn-soybean meal based diet

^b^Change in analyzed dietary NDF, percentage units

^c^As determined by sigmoidal response of phase × diet treatment means obtained from Table 3. The Cr limit of quantitation was 0.3 mg Cr/kg

^d^As determined by sigmoidal response of phase × diet treatment means obtained from Table 4. The Ti limit of quantitation was 6 mg Ti/kg

The main effect of the P1 diet on P2 fecal Ti concentration is reported in Table 4 and graphically depicted in Fig. 3b. Prior to d-16, fecal Ti was below the laboratory LOQ of 6 mg/kg fecal DM. On d-16, P2 fecal Ti for pigs fed the DDC diet in P1 was greater than for pigs fed the CS or DDGS diets, with no difference observed in P2 fecal Ti between pigs fed the CS and DDGS diets. After d-16, diets fed during P1 had no effect on P2 fecal Ti concentrations. The main effect of P2 diet on P2 fecal Ti concentration is reported in Table 4 and graphically depicted in Fig. 3c. There were no differences observed between P2 fecal Ti concentrations among pigs fed the diets for d-14 through d-16. From d-17 through d-21, pigs fed the DDC diet during P2 had a higher P2 fecal Ti concentration than pigs fed either the CS or DDGS diets, and pigs fed the CS diet had a higher P2 fecal Ti concentration when compared to pigs fed the DDGS diets.

## Discussion

Others [18–20] have reviewed criteria necessary for the use of markers in digestibility studies, but in addition to these, a critical factor for the present study was that analysis of Cr and Ti in the same diet would not interfere with the analysis of either element. Full recovery of Cr [20, 21] and Ti [22, 23] has been shown to be problematic, which was the case in our dual marker recovery experiment (Cr recovery of 77%, Ti recovery of 84%; Table 2) and animal experiment (Cr recovery of 82%, Ti recovery of 97%; Table 1) as well. The lack of any major differences in recovery of dual markers in our experiment is supported by others [24, 25] who have noted little impact of dual markers on individual marker recovery. Nonetheless, despite any potential differences in marker recovery, we believe that the data obtained in our animal experiment is valid in determining the time from which a new collection period could begin without the previous marker interfering with the results obtained in the subsequent collection period. Taken together, the literature and our data suggest that use of two markers within the digestive tract does not compromise or confound the results that we obtained in our animal experiment. We also chose to sample pigs at the same time each day to eliminate any confounding effects relative to diurnal variation in fecal composition that has been previously reported [12, 26, 27].

Numerous experiments have been conducted to describe the time of first or 5% marker appearance [4, 5, 11, 28], mean transit rate [6, 8, 28–31] or 25, 50, 80, or 95% of the marker excreted [4, 5, 7]; values which are useful in mathematical modeling of digestion [3]. This was not the focus of our experiment as we chose to only determine when P2 fecal Cr reached its minimum LOQ and when P2 fecal Ti reached 95% of its maximum because we were interested in if the previous or present diet affected when a dietary marker was completely excreted (Cr, Table 3) or stabilized (Ti, Table 4).

It is well known that the dietary fiber type and level affects rate of passage [6–8]. These effects were not, however, independent from the previous diet fed as indicated by the interaction between diets fed during P1 and P2. The current data indicate that as dietary NDF increased from P1 to P2, it took less time for P2 fecal Cr to decrease (2.6 d) or P2 fecal Ti to be maximized (2.4 d), than if NDF was decreased from P1 to P2, where it took 3.5 d for P2 fecal Cr to decrease or 3.2 d for P2 fecal Ti to be maximized. These effects were independent from feed intake in the current study because in most instances ADFI was not a significant covariate (although ADFI was still retained in the model to eliminate even minimal feed intake differences). Differences in P2 fecal marker concentration (Cr or Ti in the current study) by diet type were expected due to digestibility differences among ingredients utilized in diet formulations. With components in the diets digested to different degrees but the marker remaining undigested, subsequent Cr or Ti concentration should have changed proportionally. In the current experiment, pigs fed the diet having the greatest digestibility (DDC) resulted in the greatest fecal marker concentration, followed by pigs fed the CS diet, and lastly, by pigs fed the DDGS diet.

## Conclusions

Overall, data from the present study indicate that as the digestibility of the diets increased (i.e., decreasing dietary NDF), it took progressively longer for P2 fecal Cr to be excreted or P2 fecal Ti to be maximized (approximately 2.5 d) than if diets that were decreasing in digestibility (i.e., increasing dietary NDF) were fed (approximately 3.4 d), a difference of approximately 1 d. For pigs fed diets containing a moderate amount of DDGS or only corn and soybean meal, the complete elimination of Cr in P2 feces or maximum appearance of Ti in P2 feces was approximately 3 and 4 d, respectively. In contrast, pigs fed diets containing highly digestible ingredients such as DDC (or semi-purified diets as are used in some experimental methodologies) took longer for clearance or equilibrium, approximately 5 d in the current experiment. This information is critical to know when pigs are utilized either once or for multiple times in digestibility experiments containing inert makers, and suggest that caution must be taken so as to not have previous dietary marker addition remain in the digestive tract or feces to confound subsequent experimental results.

## Abbreviations

ADFI: Average daily feed intake
BW: Body weight
C: Corn
Cr: Chromium
CV: Coefficient of variation
d: Day
DDC: Dehulled ddgermed corn
DDGS: Distillers dried grains with solubles
h: Hour
LOQ: Limit of quantitation
NDF: Neutral detergent fiber
P1: Phase 1
P2: Phase 2
S: Soybean meal
SD: Standard deviation
Se: Standard error
Ti: Titanium
